# Machine learning algorithms in microbial classification: a comparative analysis

**DOI:** 10.3389/frai.2023.1200994

**Published:** 2023-10-19

**Authors:** Yuandi Wu, S. Andrew Gadsden

**Affiliations:** Department of Mechanical Engineering, Intelligent and Cognitive Engineering Laboratory, McMaster University, Hamilton, ON, Canada

**Keywords:** machine learning, deep learning, convolutional neural networks, transfer learning, bacterial classification

## Abstract

This research paper presents an overview of contemporary machine learning methodologies and their utilization in the domain of healthcare and the prevention of infectious diseases, specifically focusing on the classification and identification of bacterial species. As deep learning techniques have gained prominence in the healthcare sector, a diverse array of architectural models has emerged. Through a comprehensive review of pertinent literature, multiple studies employing machine learning algorithms in the context of microbial diagnosis and classification are examined. Each investigation entails a tabulated presentation of data, encompassing details about the training and validation datasets, specifications of the machine learning and deep learning techniques employed, as well as the evaluation metrics utilized to gauge algorithmic performance. Notably, Convolutional Neural Networks have been the predominant selection for image classification tasks by machine learning practitioners over the last decade. This preference stems from their ability to autonomously extract pertinent and distinguishing features with minimal human intervention. A range of CNN architectures have been developed and effectively applied in the realm of image classification. However, addressing the considerable data requirements of deep learning, recent advancements encompass the application of pre-trained models using transfer learning for the identification of microbial entities. This method involves repurposing the knowledge gleaned from solving alternate image classification challenges to accurately classify microbial images. Consequently, the necessity for extensive and varied training data is significantly mitigated. This study undertakes a comparative assessment of various popular pre-trained CNN architectures for the classification of bacteria. The dataset employed is composed of approximately 660 images, representing 33 bacterial species. To enhance dataset diversity, data augmentation is implemented, followed by evaluation on multiple models including AlexNet, VGGNet, Inception networks, Residual Networks, and Densely Connected Convolutional Networks. The results indicate that the DenseNet-121 architecture yields the optimal performance, achieving a peak accuracy of 99.08%, precision of 99.06%, recall of 99.00%, and an F1-score of 98.99%. By demonstrating the proficiency of the DenseNet-121 model on a comparatively modest dataset, this study underscores the viability of transfer learning in the healthcare sector for precise and efficient microbial identification. These findings contribute to the ongoing endeavors aimed at harnessing machine learning techniques to enhance healthcare methodologies and bolster infectious disease prevention practices.

## 1. Introduction

In the realm of healthcare, deep learning has emerged as a transformative force, particularly in the identification of microbiological species. Leveraging the power of artificial neural networks, deep learning models have revolutionized the way we approach complex problems, mimicking the intricate cognitive processes of human cognition. As the healthcare landscape evolves, data-driven solutions gain prominence. Machine Learning (ML), a subset of artificial intelligence, plays a pivotal role by enabling computers to learn autonomously from data. This departure from rule-based programming allows systems to define their own patterns and rules by learning from information. In medical contexts, this shift has ushered in a new era, managing copious amounts of data efficiently (Greenspan et al., 2016; Min et al., 2017; Xing et al., 2017; Peiffer-Smadja et al., 2020). With modern healthcare embracing rapid data acquisition, traditional labor-intensive approaches to diagnosis and prediction become conspicuously inefficient.

Classic ML algorithms have already made substantial contributions in fields like bioinformatics, utilizing regression and classification to model diagnostics. Traditional approaches involve crafting features for enhanced interpretability, with models optimizing these features in a sequential process (Jordan and Mitchell, 2015). With the advent of deep learning, novel algorithms are capable of autonomously learning intricate features, reducing the reliance on domain expertise (LeCun et al., 2015; Greenspan et al., 2016). It is a field that has seen significant success in areas such as image analysis and computer vision (Yuan et al., 2015; Druzhkov and Kustikova, 2016; Pak and Kim, 2017), whereby the ability to automatically determine and categorize important features proves to be invaluable. Currently, several types of deep neural networks exist, with each having its respective advantages and disadvantages over the other depending on the field of application. For the purposes of image classification, by far the most popular form of neural network implemented is the Convolutional Neural Network (CNN) (Rawat and Wang, 2017). CNNs are preferred for their superior computational efficiency with respect to calculations of model weights and bias values, by which the model is optimized. Several variations in the architecture of a CNN, with varying complexity and performance, have been applied to and evaluated with various datasets.

In spite of their accomplishments in image recognition tasks, deep learning architectures possess inherent limitations. These limitations stem from the substantial demand for ample high-quality data to establish prediction accuracy and robustness. Mitigating this constraint, data augmentation techniques are commonly utilized. These techniques involve modifications to the original data, aiming to enhance data volume and diversity. Additionally, recent research in image classification has embraced transfer learning. This entails repurposing pre-trained models from different datasets to evaluate smaller datasets, thereby circumventing the extensive feature extraction training process (Zhaung et al., 2020). In the realm of healthcare, substantial time and human resources are necessary to amass a substantial dataset for machine learning. Traditionally, professionals in the sector manually collect essential data using specialized tools to diagnose and identify microorganisms. Moreover, these professionals' expertise is indispensable for accurate labeling (Qu et al., 2019). Machine learning and deep neural networks present an avenue for automation, leading to reduced operational costs and heightened industry efficiency. Leveraging transfer learning and pre-trained models facilitates training deep neural networks like CNNs with fewer annotated samples, all while demonstrating nearly equivalent, if not superior, performance in classification endeavors.

This study offers a comprehensive panorama of contemporary machine learning methodologies applied in healthcare, with a specific focus on infectious disease prevention and the identification of bacterial entities. The research begins with a succinct overview of current machine learning algorithms in the healthcare sector, emphasizing their role in diagnosing and classifying bacterial species. Notably, the study incorporates a meticulous literature review that spans a range of works employing machine learning and deep learning algorithms for microbe diagnosis. The spotlight is on Convolutional Neural Networks, a pivotal choice for over a decade due to their ability to autonomously extract distinguishing features with minimal human intervention. An emerging trend of leveraging transfer learning, which repurposes pre-trained models for microbial image classification, is highlighted as well. Importantly, this research advances beyond previous reviews by not only summarizing existing research but also practically implementing and evaluating transfer learning algorithms for microbial detection using the DIBAS dataset. This approach adds a practical dimension to the study, showcasing the real-world efficacy and limitations of these techniques.

Specifically, the investigation delves into deep transfer learning algorithms' effectiveness in microscopic bacteria image classification. The study evaluates multiple pre-trained CNN networks in terms of their accuracy, precision, f1-score, convergence rate, computational complexity, and overall efficiency. The outcomes not only advance current understanding but also provide insights for optimizing future image recognition tasks related to bacterial classification. This research enhances the integration of machine learning in healthcare and contributes to refining microbial identification practices. The following report is structured as follows: Section 2 comprises a literature review elucidating prevalent machine learning and deep learning techniques presently applied within the domain of infectious disease prevention, specifically pertaining to bacterial classification. In Section 3, an introduction to the comparative analysis is provided, encompassing foundational information regarding CNNs and detailing the experimental configuration encompassing dataset usage and executed data augmentations. Section 4 examines the utilized transfer learning algorithms and their efficacy on the dataset. The study involves exploring common transfer learning models and assessing their performance on a dataset of bacterial species images. The achieved performance metrics are meticulously organized. Following this, a meticulous analysis determines the optimal architectural approach. Lastly, Section 5 encapsulates the concluding remarks of the study.

## 2. Literature survey

### 2.1. Classical machine learning techniques reviewed

The healthcare sector has witnessed notable achievements through the implementation of machine learning (ML) algorithms. Over the past decade, a multitude of novel ML systems have been devised to facilitate the decision-making process within diverse medical contexts. Classical ML techniques have garnered considerable utilization for tasks involving classification and regression, as delineated subsequently. However, these conventional ML approaches necessitate substantial human involvement in the curation of informative features—distinctive attributes or quantifiable characteristics (Greenspan et al., 2016). Noteworthy models like support vector machines (SVM), random forests, and logistic regression have demonstrated remarkable efficacy in healthcare applications, particularly in the realm of classification tasks. A concise overview encompassing the surveyed literature is presented in Table 1.

**Table 1:** Summary of ML algorithms and their application in diagnostics and classification.

**Study name**	**Year published**	**Techniques employed**	**Description**	**Reference**
Tuberculosis bacteria detection based on Random Forest using fluorescent images	2016	Random forest, linear SVM, cross validation SVM	Proposes a new RF-based tuberculosis bacilli detection method using fluorescent microscopic images and feature extraction techniques. Conducted on a dataset comprising 768 positive objects and 1,664 negative objects. The RF-based approach outperforms other popular machine learning methods, such as LinSVM and CVSVM, in terms of sensitivity, specificity, and accuracy	Zheng et al. (2016)
Diagnosing tuberculosis with a novel support vector machine-based artificial immune recognition system	2015	SVM, KNN	Introduces a hybrid system that incorporated an SVM into Artificial Immune Recognition System for diagnosing tuberculosis. The dataset used was patient epacris reports consisting of 114 positive samples for tuberculosis and 60 negative samples. Classification performance was evaluated using 10-fold cross-validation, and the proposed method achieved an accuracy of 100%, sensitivity of 100%, specificity of 100%, Youden's Index of 1 Area Under the Curve of 1, and RMSE of 0	Saybani et al. (2015)
An automated tuberculosis screening strategy combining X-ray-based computer-aided detection and clinical information	2016	Random forest, extremely randomized trees	Developed machine learning-based combination framework, evaluated on a dataset of 392 patient records from suspected tuberculosis subjects. Framework utilizes computer-aided detection scores from chest radiographs and 12 clinical features to estimate the risk of active disease. Results show that the combination framework outperforms individual strategies, achieving higher area under the receiving operating characteristic curve (0.84 vs. 0.78 and 0.72), specificity at 95% sensitivity (49% vs. 24% and 31%), and negative predictive value (98% vs. 95% and 96%)	Melendez et al. (2016)
In-vitro diagnosis of single and poly microbial species targeted for diabetic foot infection using e-nose technology	2015	KNN, linear discriminant analysis, neural networks	Developed an electronic nose technique for rapid identification of pathogenic bacteria responsible for diabetic foot infections. Using statistical approaches such as Support Vector Machine (SVM), K Nearest Neighbor (KNN), Linear Discriminant Analysis (LDA), and neural networks like Probability Neural Network (PNN), the authors identified both single and poly microbial species with up to 90% accuracy, indicating its potential as a complementary diagnostic tool for diabetic foot infections	Yusuf et al. (2015)
Prospects for clinical application of electronic-nose technology to early detection of Mycobacterium tuberculosis in culture and sputum	2006	SVM, KNN MLP	Explores the potential of gas sensor array to detect different Mycobacterium species and Pseudomonas aeruginosa in cultures and spiked sputum samples, aiming to provide a rapid and automated method for early diagnosis of respiratory infections. The device demonstrated promising results, correctly predicting culture-positive patients with 89% accuracy. The method shows a sensitivity and specificity of 89% and 91% respectively	Fend et al. (2006)
Toward automated detection, semi-quantification and identification of microbial growth in clinical bacteriology: A proof of concept	2017	Random Forest	Presents algorithms for intelligent image analysis for automated detection, semi-quantification, and identification of bacterial colonies. The algorithms demonstrated high sensitivity (97.1%) and specificity (93.6%) for microbial growth detection, accurate quantification (80.2% and 98.6% with 1 log tolerance), and identification accuracy (98.3% to 99.7% depending on bacterial species)	Croxatto et al. (2017)

Zheng et al. made comparisons between three ML classifier algorithms in the detection of Tuberculosis bacteria from fluorescent images. Images were captured from processes such as fluorescent microscopy and or bright field microscopy (Zheng et al., 2016). To extract feature representations of the bacteria object, diverse techniques were employed. Notably, Hu Moment Invariants, a set of seven parameters derived from central image moments, were used due to their resistance to image transformations (Huang and Leng, 2010). Additional methods included geometric shape properties and histograms of oriented gradients, effectively capturing the distinctive rod-like morphology of Tuberculosis bacteria. The study focused on binary classification, employing the aforementioned algorithms. Of which, the Random Forest classifier consistently showed higher performance than SVM methods with regard to sensitivity, specificity and accuracy metrics. In relation to computational complexity and efficiency, it is observed that the cross-validation SVM approach exhibited the highest level of complexity. The computation times associated with this method were approximately two orders of magnitude greater than those of the second most intricate algorithm, namely the Random Forest.

Saybani et al. (2015) developed a novel method of Mycobacterium Tuberculosis diagnosis in patients with the use of an SVM artificial immune recognition system (Saybani et al., 2015). Artificial immune systems simulate the human immune system, utilizing prior memory to identify and recognize patterns. Research done in this area involves Watkins' supervised algorithm, the Artificial Immune Recognition System (Watkins, 2001), and Brownlee's modification to the previous system, Artificial Immune Recognition System 2 (Brownlee, 2005). The group innovated on this model further by providing an alternate, and more effective SVM classifier with radial basis function kernel as opposed to the KNN classifier used in previous studies. The dataset involves 175 samples from a patient pool afflicted with Mycobacterium Tuberculosis and features various statistical measurements of key biological bodies or chemical compounds, the presence of various symptoms, patient biological information, as well as whether the patients tested positive for Mycobacterium Tuberculosis. The classification criterion involves the minimization of the mean squared error. A 10-fold cross-validation was performed. Alternate measures of performance involve sensitivity and specificity, Youden's Index, and Area Under the Curve (AUC). Overall, the researchers attained a 100% accuracy, sensitivity, and specificity in their outcomes, alongside an absence of mean squared error.

Regarding tuberculosis diagnosis, Melendez et al. (2016) conducted research in this area. Their study involved the development of an automated system that collects patient data using X-ray-based computer-aided detection in conjunction with clinical information (Melendez et al., 2016). Feature selection was determined from both X-ray imaging, as well as patient's clinical data, and selected utilizing the minimum redundancy maximum relevance algorithm. The classification was performed using a combination of the Random Forest algorithm and the Extremely Randomized Trees (ERT). Both methods share commonalities through their utilization of randomization and their categorization as tree ensemble methods. However, a distinguishing feature ERTs compared to Random Forest lies in the nature of decision tree splits. ERT employs random splits for decision trees, in contrast to the deterministic splits employed by Random Forest. The optimization process encompassed adjustments to the count of decision trees and the maximal depth of these trees. The learning process was performed and evaluated using 10-fold cross-validation. Evaluation metrics were accuracy, sensitivity specificity, area under the curve and negative predictive value. The study evaluated various metrics including accuracy, sensitivity, specificity, area under the curve, and negative predictive value. The combined dataset framework showed superior accuracy and specificity compared to using X-ray imaging or clinical data alone. The results of the reviewed study demonstrated that the combination framework outperforms individual strategies in terms of AUC (0.84 vs. 0.78 and 0.72), specificity at 95% sensitivity (49% vs. 24% and 31%), and negative predictive value (98% vs. 95% and 96%).

Yusuf et al. (2015) also performed classification of bacteria through their analysis of Volatile Organic Compounds (VOCs) with electronic noses (Yusuf et al., 2015). In their study, the diagnosis of Mycobacterium tuberculosis was performed via analysis of multivariate data through the Principal-component analysis (PCA), discriminant function analysis (DFA), and a back propagation neural network. Overall, the system was capable of predicting 89% of culture-positive patients.

Analysis of VOCs was also the approach utilized by Fend et al. (2006) in the rapid diagnosis of microbes causing diabetic foot infection (Fend et al., 2006). The group utilized a combination of Linear Discriminant Analysis (LDA) with alternative classification algorithms such as SVM, KNN, and the Multi-Layer Perceptron (MLP) neural network model on data from solid phase microextraction mass spectroscopy. The hybrid system outlined demonstrates improved robustness and performance over conventional methods, accurately forecasting culture-positive patients with an 89% precision rate. The approach displays sensitivity and specificity rates of 89% and 91%, respectively.

The Random Forest algorithm was employed by Croxatto et al. (2017) in their study to undertake classification, quantification, and identification of microbial entities. The study employed the Boruta Algorithm to select feature inputs due to its balanced performance-computational speed trade-off. Notably, the automated classification accuracy varies between 98.3% and 99.7%, contingent on the bacterial species.

### 2.2. Deep learning techniques in clinical diagnostics and classification of microbial species

In contrast to conventional techniques in machine learning, deep learning methodologies are highly regarded for their inherent capacity to autonomously discern pertinent features intrinsic to a given dataset. In the domain of diagnostic procedures and taxonomical categorization of microorganisms, an assortment of deep learning approaches has been adeptly harnessed. Among these methodologies, neural networks, inspired by the cognitive information processing paradigm of biological neural ensembles, have garnered conspicuous prominence. Evidencing their prowess, neural networks have demonstrated adeptness in furnishing precise approximations to intricate problem sets, registering achievements commensurate with those of domain experts (Sarvamangala and Kulkarni, 2022). Diverse instantiations of neural network architectures have been scrutinized within the purview of the healthcare domain. One paradigmatic manifestation, denoted as the Multilayer Perceptron (MLP) model, entails an arrangement of neuron-stratified tiers through which informational propagation occurs unidirectionally. In the more contemporary landscape, the Convolutional Neural Network (CNN), initially conceived by LeCun et al. (2015), has garnered conspicuous traction. The salient attributes of CNNs encompass heightened computational efficiency along with the proficiency to effectually ameliorate and condense the multidimensionality characterizing image datasets. Inclusive of these developments, an encapsulation of pertinent scholarly works is succinctly tabulated in Table 2 for comprehensive reference.

**Table 2:** Summary of deep learning algorithms and their application in diagnostics and classification.

**Study Name**	**Year published**	**Techniques employed**	**Description**	**Reference**
FT-IR Hyperspectral Imaging and Artificial Neural Network Analysis for Identification of Pathogenic Bacteria	2018	Two level MLP model trained with Resilient Back Propagation	Employs FT-IR hyperspectral imaging and optimized artificial neural networks for rapid and cost-effective identification of pathogenic bacteria, involving machine learning-based image segmentation for taxonomic resolution. The neural network classifiers are trained using spectral data from biological replicates, including spectral quality assessment and PHB interference removal, utilizing NeuroDeveloper software and rprop learning algorithm	Lasch et al. (2018)
Fourier Transform Infrared Spectroscopy for Rapid Identification of Nonfermenting Gram-Negative Bacteria Isolated from Sputum Samples from Cystic Fibrosis Patients	2008	Two level MLP model trained with back propagation	Introduces a novel method utilizing FTIR combined with ANNs for the rapid identification of nonfermenting gram-negative rods, including various bacterial species prevalent in cystic fibrosis patients, achieving high identification success rates of 98.1% for broader categories and 93.8% for specific Burkholderia cepacia complex species	Bosch et al. (2008)
Rapid identification of pathogenic bacteria using Raman spectroscopy and deep learning	2019	CNN, SVM, logistic regression	Presents a deep learning-based method for accurately identifying 30 common bacterial pathogens using Raman spectroscopy, achieving isolate-level accuracies over 82% and antibiotic treatment identification accuracies of 97.0 ± 0.3%, including distinguishing between MRSA and MSSA with 89 ± 0.1% accuracy, validated on clinical isolates from 50 patients, with potential for culture-free pathogen identification and antibiotic susceptibility testing using minimal spectra	Ho et al. (2019)
Bacterial colony counting with convolutional neural networks in digital microbiology imaging	2017	CNN trained with SGD, SVM, random forest	Explores automated bacterial colony counting using machine learning, comparing a handcrafted feature-based SVM approach with a CNN-based deep learning method, with the CNN approach significantly outperforming the handcrafted features in bacterial load estimation, presenting an overall accuracy of 79.5%	Ferrari et al. (2017)
Accurate prediction of blood culture outcome in the intensive care unit using long short-term memory neural networks	2018	MLP, CNN	Presents a novel approach using a bidirectional long short-term memory neural network to predict the outcome of blood culture tests based on nine clinical parameters measured over time, achieving high predictive accuracy (AUC: 0.99, precision-recall AUC: 0.82) and outperforming non-temporal machine learning models, offering potential for early detection of bloodstream infections in ICU patients	Kuhl and Giardina (1982)
Tuberculosis disease diagnosis using artificial neural networks	2010	MLP trained with Backpropagation and Levenberg Marquardt algorithm	Presents a study on tuberculosis diagnosis using multilayer neural networks, comparing two such network structures (one with one hidden layer and the other with two hidden layers) and a general regression neural network using Levenberg-Marquardt algorithms for training; achieving a highest classification accuracy of 94.88%	Er et al. (2010)
Neural networks: an application for predicting Smear negative pulmonary tuberculosis	2007	MLP trained with Backpropagation	Introduces a neural network model for diagnosing smear-negative pulmonary tuberculosis based solely on symptoms and physical signs, achieving a 77% correct classification rate on a test sample of 136 patients	Santos et al. (2007)

The field of spectroscopy is a study of the interaction of radiation with matter, encompassing absorption, emission, reflection, transmission, and scattering of light or other forms of electromagnetic radiation by different materials. Within this area of study, (Lasch et al., 2018) and (Bosch et al., 2008) made use of characteristic bacterial absorption and transmissions of five different microorganisms as single or mixed cultures of infrared light for the purposes of pathogenic bacteria identification. The technique employed to capture bacteria information is known as the Fourier transformed infrared spectroscopy (FTIR). Both studies feature MLP networks trained using the resilient back-propagation learning algorithm to classify the spectra generated by the FTIR. Multiples of such networks were utilized to form a modular hierarchical network, in which the top-level network categorized bacteria in a broad sense.

In the paper by Lasch et al. (2018), the MLP network consists of the most basic 3 layers: an input, an output and one hidden layer. A top-level MLP network performs the task of automatic spectral quality tests. Whereas the following network performs classification on spectra corresponding to 11 strains of bacteria, previously classified as positive quality by the top-level network. Through this basic framework, an accuracy of 954 out of 1,274 pixel spectra, or around 75% of the data was correctly classified. The study by Bosch et al. (2008) also consists of an MLP network and may be summarized as follows: A top-level neural network classified species of P. aeruginosa, *S. maltophilia, Achromobacter xylosoxidans, Acinetobacter spp., R. pickettii*, and *Burkholderia cepacia* complex bacteria. The model consists of an input layer of size 60, 2 hidden layers, and an output layer of size 6. An accuracy of 98.1% was achieved by the top-level network. The second neural network serves to classify 4 specific sub-strains of bacteria: *B. cepacia, B. multivorans, B. cenocepacia*, and *B. stabilis*, under *Burkholderia cepacia* complex bacteria, and utilizes a similar model with an output layer of size 4 instead. This model achieved an accuracy of 93.8%.

In another study by Ho et al., a CNN architecture was employed for the rapid classification of pathogenic bacteria through Raman Spectroscopy (Ho et al., 2019). Through this approach, Ho et al. effectively classified 30 species of pathogenic bacteria, with an average classification accuracy of 82%. The CNN architecture consists of 25 convolutional layers. Each layer takes a one-dimensional vector as input, representing the spectra. An important innovation within this study involves the omission of pooling layers in the CNN design. Instead, the approach incorporates strided convolutions to maintain precise spectral point positioning of interest. Baseline comparisons were made with classical ML algorithms such as the SVM and Logistic regression. The study's results indicate that CNNs exhibited superior performance compared to conventional ML methods, with SVM having a 75.7% accuracy, and Logistic regression having a 74.9% accuracy.

In the research conducted by Ferrari et al. (2017), CNNs and SVMs were employed to execute the tasks of bacteria colony quantification and classification. Captured images are segmented utilizing SVM with Radial Basis Function kernels to generate a binary mask of colonies, effectively forming the input data. The CNN is trained using Stochastic Gradient Descent (SGD) with momentum, and consists of 4 convolutional layers, followed by a fully connected layer and a soft-max layer. A dropout of 75% was utilized on the outputs of the fully connected layer, to reduce overfitting. In all, CNN was able to achieve an accuracy of 91.5%. For the SVM classifier, features were extracted using Elliptic Fourier Descriptors (Kuhl and Giardina, 1982). A Random Forest algorithm was utilized for feature selection, and features were classified with the SVM approach with Radial Basis Function kernels. An accuracy of 79.5 % was reported.

In the realm of diagnostic methodologies, Er et al. (2010) have made a notable contribution through their investigation into the potential applications of neural networks for Tuberculosis diagnosis (Er et al., 2010). Specifically, their study revolves around the utilization of a Multilayer Perceptron (MLP) model. The study involves a comparative analysis of outcomes derived from two distinct architectures of the MLP. The experimental dataset encompassed 150 samples derived from patient records. Each of these samples comprised 38 essential attributes, which encompassed a range of biological data, patient-reported symptoms, as well as quantified concentrations of various compounds including cholesterol, calcium, blood urea nitrogen, chlorine, among others. The architectural configuration of the MLP model incorporates an input layer of dimension 38, succeeded by either one or two hidden layers comprising 50 neurons each. The terminal layer, constituting the output, exhibits a dimensionality of 2. This specific two-dimensional output signifies the binary classification associated with the presence or absence of Mycobacterium Tuberculosis. Neural networks are trained with two different algorithms: backpropagation, and the Levenberg-Marquardt algorithm (Hagan and Menhaj, 1994). In general, the Levenberg-Marquardt algorithm converges faster than that of traditional gradient descent algorithms, however, gradient descent has been shown to be more stable and robust (Amin et al., 2011). Overall, the researchers determined that increasing hidden layers of neurons also increases the performance of the network. It was determined that for this application, the Levenberg-Marquardt algorithm demonstrates a higher level of performance in comparison to the SGD method, with the highest accuracy of 95.08% when utilized to train an MLP with two hidden layers.

On the same topic, Santos et al. (2007) also utilized neural networks in their diagnosis of smear-negative pulmonary Tuberculosis. The network architecture comprises a three-layer Multilayer Perceptron (MLP) consisting of an input layer, a hidden layer, and an output layer. This design is influenced by the model introduced in (Haykin, 1994). The optimal neuron count within the hidden layer is identified as four. Empirical experiments demonstrate that introducing additional neurons, for the context of this study, leads to issues such as overfitting and reduced generalization. The dataset encompasses 136 patient samples. Among these, three distinct sets of variables are extracted, encompassing patient biological data and symptoms, which collectively form the input vector. In the case of variables, binary attributes are encoded as 1 or −1, while qualitative attributes are coded as follows: 1 for presence, 0 for disregard, and −1 for absence. The output of the network consists of a solitary neuron, and its activation corresponds to the estimated likelihood of Tuberculosis presence. Metrics for evaluation of network performance included accuracy, sensitivity and specificity, achieving a 77%, 87%, and 71% in the aforementioned fields respectively.

Deep learning methodologies, particularly neural networks, have gained prominence in the healthcare domain for their ability to autonomously discern relevant features within complex datasets. In the context of bacterial identification and diagnostics, neural networks have been employed to analyze data from various spectroscopic techniques. In studies using Fourier-transformed infrared spectroscopy (FTIR), Multilayer Perceptron (MLP) networks were trained to classify bacterial spectra. These studies achieved high accuracy, with one achieving 75% and another achieving 98.1% accuracy for broad bacterial categorization. In contrast, Convolutional Neural Networks (CNNs) were employed for rapid classification of pathogenic bacteria through Raman spectroscopy, achieving an average accuracy of 82%. Compared to classical machine learning algorithms like SVM and logistic regression, CNNs outperformed them, highlighting the effectiveness of deep learning in this context.

In bacterial colony quantification and classification, a combination of CNNs and SVMs was used, achieving an accuracy of 91.5% for CNN and 79.5% for SVM. The dataset in this study was segmented using SVM, and CNNs were trained using stochastic gradient descent with momentum. Additionally, in tuberculosis diagnosis, MLP models were employed to analyze patient data and biological attributes, achieving high accuracy levels of up to 95.08%. The choice of training algorithm, such as backpropagation or the Levenberg-Marquardt algorithm, played a crucial role in determining the network's performance. Another study focused on diagnosing smear-negative pulmonary tuberculosis using a three-layer MLP, achieving an accuracy of 77% with sensitivity and specificity of 87% and 71%, respectively. Overall, deep learning techniques, especially neural networks, have demonstrated their effectiveness in bacterial identification, spectroscopy analysis, and disease diagnosis within the healthcare sector, often outperforming traditional machine learning approaches. These methods leverage the capability of deep learning to automatically extract intricate patterns and features from complex datasets, contributing to improved accuracy and efficiency in medical applications.

### 2.3. Transfer learning techniques in clinical diagnostics and classification of microbial species

Transfer learning is a methodology that centers on leveraging acquired insights from one task to address a closely aligned task. Contemporary advancements in transfer learning frameworks have enabled several researchers to enhance the efficacy of systems designed for the categorization of microbiological entities. An overview encompassing the body of literature employing transfer learning techniques within the realm of bacteria classification is presented in Table 3.

**Table 3:** Summary of Transfer Learning Algorithms and their application in diagnostics and classification.

**Study Name**	**Year published**	**Techniques employed**	**Description**	**Reference**
Novel neural network application for bacterial colony classification	2018	CNN trained with SGD, AlexNet, SVM	Presents an automatic program using deep convolutional neural networks (CNNs) for classifying bacterial colony morphology, achieving a 73% overall classification accuracy and up to 90% accuracy and specificity for individual bacterial species, demonstrating potential for efficient bacterial pre-screening	Huang and Wu (2018)
Deep learning approach to bacterial colony classification	2017	AlexNet, VGG-D, VGG-VD	Employs deep Convolutional Neural Networks for texture analysis of bacterial images, utilizing Support Vector Machine or Random Forest for classification, achieving a recognition accuracy of 97.24 ±1.07% on a new dataset of 660 images containing 33 bacterial genera and species	Zieliński et al. (2017)
Deep bacteria: Robust deep learning data augmentation design for limited bacterial colony dataset.	2019	CNN	Introduces a deep neural network architecture for classifying bacterial colonies, utilizing data augmentation to address limited dataset size, achieving a testing accuracy of 98.22%	Khalifa et al. (2019)
Deep learning based bacteria classification	2018	AlexNet, VGGNet	Presents a study on deep learning-based bacteria classification using the DIBaS dataset, employing VggNet and AlexNet training models in MATLAB; achieving classification accuracies of 98.25% with VggNet and 97.53% with AlexNet for 33 different bacteria species.	Nasip and Zengin (2018)
Small-scale depth wise separable convolutional neural networks for bacteria classification	2021	CNN	Presents a method for automated classification of 33 bacteria strains using a compact five-layer depthwise separable convolutional neural network architecture, achieving a recognition accuracy of 96.28% on a dataset of 6,600 images, with low computational cost suitable for limited-resource devices	Mai and Ishibashi (2021)
Bacterial colony classification using atrous convolution with transfer learning	2021	VGG16	Presents an atrous convolution based network with transfer learning for automated bacterial colony classification using deep neural networks, achieving high accuracies of 95.06% training, 93.38% validation, and 94.85% test accuracy on a dataset of 660 bacterial colonies with 33 classes	Patel (2021)
An automated deep learning approach for bacterial image classification	2019	ResNet-50	Presents an automated deep learning approach using the ResNet-50 pre-trained CNN architecture for classifying bacteria species from microscopic images, achieving a high average classification accuracy of 99.2%	Talo (2019)
An enhanced classification of bacteria pathogen on microscopy images using deep learning	2021	DenseNet-201	Presents an enhanced classification technique for bacterial pathogen identification using the DensNet201 pre-trained CNN architecture with transfer learning and freeze layer technique, achieving an accuracy of around 99.24%	Akbar et al. (2021)
Efficient deep learning architectures for fast identification of bacterial strains in resource-constrained devices	2021	EfficientNetMobileNet V2, MobileNet V3, SqueezeNet	Introduces twelve fine-tuned deep learning architectures for bacterial classification using digital images, incorporating a novel artificial zoom-based data augmentation technique that significantly enhances performance, achieving top-1 accuracy scores up to 0.9738, evaluated through cross-validation	García et al. (2021)
Efficient detection of longitudinal bacteria fission using transfer learning in deep neural networks	2021	ResNet18	Presents an automated solution for classifying the longitudinal division of bacteria employing ResNet. Transfer learning is applied to train a binary classification model that identifies bacterial division, achieving high test accuracy (99%) with pre-trained models and stabilizing by epoch 5, contrasting with a non-pre-trained model stabilizing by epoch 12; this approach eliminates the need for manual classification and benefits from data augmentation and pre-trained networks	Garcia-Perez et al. (2021)
AGAR a microbial colony dataset for deep learning detection	2021	Faster R-CNN, Cascade R-CNN, ResNet, ResNeXt, HRNet	Presents an exhaustive analysis of various deep neural networks for object detection in microbial colonies, demonstrating the superiority of the Cascade RCNN with HRNet backbone, achieving low counting errors of 4.92% and 3.81% on different subsets, and mAP scores between 49.3% to 59.4% for detection	Majchrowska et al. (2021)
A transfer learning-based approach with deep CNN for COVID-19- and pneumonia-affected chest X-ray image classification	2021	VGG19	Applies transfer learning using the pre-trained VGG-19 architecture to classify COVID-19, Pneumonia, and Healthy cases from chest X-ray images, achieving a test dataset accuracy of 97.11%, average precision of 97%, and average recall of 97%	Chakraborty et al. (2022)

Huang et al. employed neural networks for the purpose of classifying 18 strains of prevalent human pathogenic bacteria based on their morphological characteristics (Huang and Wu, 2018). The utilized approach involved the implementation of a Convolutional Neural Network (CNN) trained with the Stochastic Gradient Descent (SGD) algorithm incorporating momentum. This network's performance was assessed in comparison to a pre-trained CNN known as AlexNet. Notably, relevant features were extracted from input images at the fully-connected layer of the CNN, subsequently forming a matrix of features. In conjunction with a pre-labeled classifier matrix, an SVM may be constructed to classify each strain. Through analysis, the two approaches shared comparable results in terms of accuracy. In terms of sensitivity, AlexNet exceeds that of conventional CNNs due to its highly compressed convolutional layers.

Zieliński et al. also evaluated the classification capabilities of several machine learning models in their study (Zieliński et al., 2017). The group employed the Digital Images of Bacterial Species (DIBaS) dataset. The dataset contains over 660 digital images of 33 strains of bacteria. Through their study, Zieliński et al., have, for the first time, made the DIBaS dataset publicly available, leading to a plethora of follow-up experimentations and studies by other authors in the field. For their study itself, Zieliński et al. utilized a variety of techniques in tandem. CNNs such as AlexNet, VGG-D, and VGG-VD were employed in conjunction with Dense SIFT to obtain local descriptors. Descriptors are refined with the Fisher vector and pooling encoders to obtain the feature vectors from images belonging to the DIBaS dataset. Classification is performed with Support Vector Machines (SVM), or the Random Forest technique, with an overall classification accuracy of 97% achieved.

Owing to the constrained data availability within the DIBaS Dataset, Khalifa et al. (2019) introduced a transfer learning paradigm utilizing the architectural framework of AlexNet, augmented by data enrichment strategies (Khalifa et al., 2019). Traditional neural networks demand a substantial dataset for effective training, thereby encountering challenges of overfitting or underfitting in cases where dataset magnitude falls short of prescribed criteria. To further enhance the amount and variety of data made available to deep learning models, Khalifa et al. (2019) employed data augmentation techniques to increase the size of the DIBaS dataset to a sample of 6,660 images for the training set, and 5,940 images for the validation set. Overall, the group was able to achieve an accuracy of 98.22% with their architecture.

Nasip and Zengin (2018) also utilize the AlexNet CNN architecture to classify 33 species of bacteria in the DIBaS dataset. The study employed the Visual Geometry Group Network (VGGNet). The network incorporates increased depth within the conventional Convolutional Neural Network (CNN) architecture, aimed at enhancing overall performance. Typical structures of VGGNet consist of 16 or 19 convolutional layers and max pooling. The activation function utilized in the network is the ReLU function. In all, Nasip and Zengin were able to achieve a top-1 accuracy of 97.53% for AlexNet and a 98.25% accuracy for VGGNet.

Mai and Ishibashi (2021) introduced an innovative compact CNN architecture designed for classification tasks utilizing the DIBaS dataset. The proposed model employs a concise five-layer structure, comprising slightly over 3.23 million parameters, which notably contrasts with the parameter counts of contemporary leading models. Notably, despite its streamlined design, the model devised by Mai and Ishibashi attains a remarkable peak accuracy of 96.28%.

In a related study, Patel conducted a parallel analysis on the DIBaS dataset, utilizing a modified iteration of the VGG16 neural network architecture (Patel, 2021). The researchers integrated atrous convolutions, also known as dilated convolutions, into their model. The extent of dilation was regulated through the dilation rate parameter, affording the flexibility to stipulate intervals between convolution filter values. The adoption of atrous convolution filters facilitated a broader receptive field, amplifying spatial coverage without significant increments in computational burden. Positioned as the terminal layer within the cascade of feature extraction convolutional layers in the VGG16 network, the atrous convolution layer preceded the subsequent fully connected layers. This configuration culminated in an achieved classification accuracy of 94.85%.

Talo employed transfer learning for the classification of bacterial species, utilizing the ResNet-50 architecture developed in He et al. (2016) in their paper on “Deep residual learning for image recognition.” He et al. (2016) demonstrated the existence of an optimal depth threshold for convolutional neural networks (CNNs) to prevent accuracy degradation. They introduced the concept of skip connections, which enable the incorporation of prior layer outputs into subsequent layers, facilitating smoother information propagation within the network. This innovation allows for the training of significantly deeper neural networks while maintaining accuracy. Through the ResNet-50 model, Talo (2019) was able to achieve a validation accuracy of 99.2% through 5-fold cross-validation, and an average accuracy of 99.12%(Talo, 2019).

Akbar et al. proposed a transfer learning model centered around the DenseNet-201 pre-trained CNN model for the classification of pathogenic bacteria species (Akbar et al., 2021). A set of 40,000 images containing six species of bacteria were evaluated with the DenseNet-201 model. Furthermore, for the purpose of benchmarking, the utilization of both the ResNet-50 and VGG-16 models was also incorporated into the analysis. Overall, it was determined that the DenseNet-201 Model boasted superior accuracy and performance with regards to the other two models, at an accuracy of 99.24%.

García et al. performed a similar bacteria classification task in their study “Efficient Deep Learning Architectures for Fast Identification of Bacterial Strains in Resource-Constrained Devices” (García et al., 2021). The study evaluated the performance of 4 separate transfer learning models on the classification of the Digital Image of Bacterial Species dataset. The learning models employed in this study were the EfficientNet model, MobileNet V2 and MobileNet V3, and SqueezeNetThe research team also utilized data augmentation by implementing various cropping techniques to decompose the initial images into multiple smaller sub-images. Through their experimentation, García et al. concluded that MobileNet V3 was superior in terms of accuracy, with a top-1 accuracy rating of 97.38%.

In their research, (Garcia-Perez et al., 2021) devised a transfer learning framework employing the ResNet-18 architecture. The investigation centered on quantifying longitudinally dividing bacterial species, exemplified by *Candidatus Thiosymbion oneisti*. Through this transfer learning paradigm, Garcia-Perez et al. successfully engineered a binary classification model with the capacity to effectively discern bacterial cells in the midst of division, achieving a remarkable accuracy of up to 99.75%.

In the study by Majchrowska et al. (2021) the group contributed a set of 18,000 images of five species of both single and mixed culture micro-organisms for the purpose of building a deep learning network (Majchrowska et al., 2021). The dataset, known as the Annotated Germs for Automated Recognition (AGAR) dataset, is comprised of five species from different bacterial groups, namely, five representatives from different bacterial groups, namely: *S. aureus* subsp. *aureus* ATCC 6538*, B. subtilis* subsp. *spizizenii* ATCC 6633, *P. aeruginosa* ATCC 9027, *E. coli* ATCC 8739, and *C. albicans* ATCC 10231. The authors further identified and classified each sample imaged as countable, uncountable, and empty. In total 11,270 images were classified as countable, 4,513 images as uncountable, and the remaining as empty. These classifications were further separated by image quality, with images separated based on their quality into the subgroups bright (2,088 images), dark (8,560 images), vague (971 images), and lower-resolution (5,830 images). The countable class, containing 12,270 images, is further examined by experts in the field with regard to colony location and specific species of bacteria. In total, 336,442 colonies of the five microbial species distributed over the countable class were labeled. Additionally, Majchrowska et al. (2021) developed Convolutional Neural Network (CNN) models to accurately classify and count bacterial colonies within the AGAR dataset. The basis for these models stemmed from the work of Girshick et al. (2014), who introduced the concept of Region-Based Convolutional Neural Networks (R-CNN) with a focus on object detection. In their study, Majchrowska et al. explored two R-CNN variants: Faster R-CNN and the Cascade R-CNN models. These models were rigorously evaluated alongside pre-trained models like ResNet, ResNeXt, and HRNet. Evaluation metrics included the Mean Absolute Error and symmetric Mean Absolute Percentage Error, which collectively demonstrated that both network architectures achieved similar levels of accuracy, with errors as low as 4.92%. This exceptional performance was attributed to the comprehensive AGAR dataset, allowing the models to generalize effectively to new data, marking a substantial advancement in the field.

Transfer learning has also been applied to detect and classify cases of COVID-19 virus and pneumonia in patients via images from chest X-rays (Chakraborty et al., 2022). Chakraborty et al. (2022) performed the study in their evaluation of a set of 3,979 chest X-ray images. The dataset is comprised of 1184 images from patients afflicted with the COVID-19 virus, 1,294 images from those afflicted with Pneumonia, and 1,319 images from healthy patients. The VGG19 pre-trained model was employed, with the last three fully connected layers replaced by a single layer composed of three neurons to represent the available classes. The classifier identifies individuals that are healthy, individuals afflicted with pneumonia, or individuals afflicted with COVID-19 from features extracted. An overall accuracy of 97% was achieved on the dataset.

## 3. Comparative analysis of existing networks

The utilization of machine learning algorithms within the healthcare sector holds significant potential, particularly concerning the identification and categorization of bacterial species. As an array of deep learning algorithms becomes increasingly accessible, a critical evaluation of their efficacy and computational performance becomes imperative, facilitating the selection of optimal strategies for specific tasks. Bridging the gap between the theoretical insights gained from the literature review conducted prior and the real-world results of a comparative analysis, the following sections aim to deliver a comprehensive overview of contemporary applications of deep transfer learning algorithms within the domain of microscopic bacteria image classification. The primary focus lies in their capacity to accurately categorize digital representations of bacterial species. In pursuit of this objective, an assessment of diverse pre-trained CNNs was conducted, encompassing crucial performance metrics such as accuracy, precision, F1-score, convergence rate, computational complexity, and overall efficiency of the network. The present study seeks to elucidate the effectiveness and suitability of deep transfer learning algorithms in the realm of bacterial species classification through an analysis of the findings. The insights garnered herein not only bridge the gap between theory and practice but also hold the potential to form a foundational basis for future advancements in the domain of medical image recognition, thereby contributing to the refinement of precise and efficient diagnostic tools tailored for bacterial classification. The following sections offer a detailed explanation of the complexities within the study, including an analysis of the dataset used for examination, the contextual framework that forms the foundation of CNNs, and the methodological structures utilized during the course of the experimentation phase.

### 3.1. Dataset evaluated

#### 3.1.1. Dataset description

**Figure 1:**
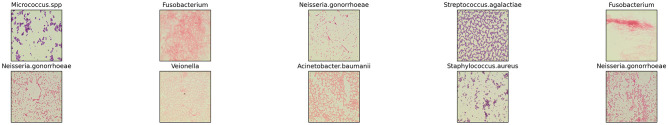
Sample of images of bacterial species from DIBaS dataset (Zieliński et al., 2017).

Dataset utilized for evaluation in this study is the Digital Image of Bacterial Species (DIBaS) dataset, published by Zieliński et al. in their study: “Deep learning approach to bacterial colony classification” (Zieliński et al., 2017). The dataset consists of 33 classes, representative of the species of bacteria captured. Each class consists of, on average, a set of ~20 digital images. A sample of ten labeled images from the DIBaS dataset is provided in Figure 1. Images within each class depicts the respective species of bacteria after Gram staining. Samples were captured with the Olympus CX31 Upright Biological Microscope and the SC30 camera. In total, 689 images of size 2,048 × 1,532 pixels were acquired. For reference, species names, and the number of available digital images of species are summarized in Table 4.

**Table 4:** Summary of DIBaS dataset.

**Species name**	**No. images**	**Species name**	**No. images**
*Acinetobacter. baumanii*	20	*Lactobacillus. plantarum*	20
*Actinomyces. israeli*	23	*Lactobacillus. reuteri*	20
*Bacteroides. fragilis*	23	*Lactobacillus. rhamnosus*	20
*Bifidobacterium. spp*	23	*Lactobacillus. salivarius*	20
*Candida. albicans*	20	*Listeria. monocytogenes*	22
*Clostridium. perfringens*	23	*Micrococcus. spp*	21
*Enterococcus. faecalis*	20	*Neisseria. gonorrhoeae*	23
*Enterococcus. faecium*	20	*Porfyromonas. gingivalis*	23
*Escherichia. coli*	20	*Propionibacterium. acnes*	23
*Fusobacterium*	23	*Proteus*	20
*Lactobacillus. casei*	20	*Pseudomonas. aeruginosa*	20
*Lactobacillus. crispatus*	20	*Staphylococcus. aureus*	20
*Lactobacillus. delbrueckii*	20	*Staphylococcus. epidermidis*	20
*Lactobacillus. gasseri*	20	*Staphylococcus. saprophiticus*	20
*Lactobacillus. jehnsenii*	20	*Streptococcus. agalactiae*	20
*Lactobacillus. johnsonii*	20	*Veionella*	22
*Lactobacillus. paracasei*	20		

It is essential to acknowledge the limitations associated with relying solely on the DIBaS dataset for assessing the effectiveness of our proposed method. While our approach has demonstrated promising results within the context of the DIBaS dataset, its performance could potentially differ when applied to other datasets that encompass distinct characteristics and challenges. Various factors contribute to this variation, including differences in image quality, lighting conditions, staining techniques, and bacterial growth patterns among different datasets. Additionally, the DIBaS dataset might not encompass the full spectrum of bacterial species diversity that exists in real-world scenarios, thereby limiting the generalizability of our method to broader bacterial classifications. Therefore, caution should be exercised when extrapolating the success observed on the DIBaS dataset to real-world applications where the dataset's unique attributes might not align with the complexities encountered in other datasets. To address this limitation and enhance the robustness of our proposed method, future research should include evaluation on diverse datasets to ensure its adaptability and effectiveness across a wider range of bacterial species and imaging conditions.

#### 3.1.2. Data augmentation

Data augmentation is the artificial inflation of data quantity via the generation and concatenation of new data to the original dataset. New data is generated from original data and involves minor alterations, such as geometric transformations or image color alterations. Overall, the objective of data augmentation is to increase data quantity and data diversity. In this study, due to the limited data available (689), data augmentation was performed to increase the amount and variety of training data made available to the deep learning network for increased robustness and performance. Data augmentation is performed using the transform method provided by the TorchVision Library. In total, from the original 689 images, data augmentation was performed to form a combined dataset of 5512 images. Transformations utilized are as follows: Horizontal flip, application of random rotation was applied, application of random image shear and scale, and translations. In addition to this, image brightness and saturation are varied through the color jitter function, contrast through the auto contrast function, and image hue through the invert function. Further to the aforementioned transforms, all images are resized to a size of 224 lengthwise pixels by 224 widthwise pixels by 3 color channels. The resizing operation is performed by means of Bilinear interpolation (Smith, 1981) to match the input size for the transfer learning models utilized.

### 3.2. Convolutional neural networks background

**Figure 2:**
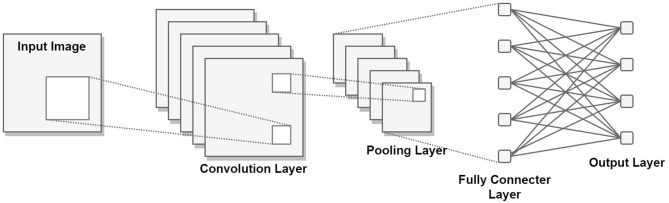
General architecture of a Convolutional Neural Network.

Neural networks, at their core, consist of substantial quantities of artificial neurons interconnected to form a network. This network structure gives rise to various types of neural networks. Among these architectures, a specific design of significance to the present study is the Convolutional Neural Network (CNN). The CNN model encompasses multiple layers of neurons. Particularly in tasks related to image classification, these layers can be categorized into two groups: the feature extraction layers and the classifier layers. Each layer takes as input the vector output from the preceding layer, subject to specific mathematical operations determined by the layer's nature. With every layer of transformation, a progressive level of abstraction is introduced to the representation. The CNN model, functioning as a form of supervised learning, finds utility when ample labeled data is available, following the insights elucidated by LeCun et al. (2015). A visual representation of this model is accessible in Figure 2.

CNNs are typically composed of convolution layers, nonlinear layers, pooling layers, and fully connected layers. In the following section, the functionality and operation of the aforementioned layers will be discussed. The convolutional layer functions as the overall feature extractor. In each convolutional layer, a weight vector is or convolved across data to produce a feature map, or matrices of locally weighted sum. In accordance with LeCun's definition (LeCun et al., 2015), the convolution action may be given mathematically as:


(1) $$\begin{array}{l} S\left(i,j\right)=\left(I^{*}k\right)\left(i,j\right)=\underset{m}{\sum }\underset{n}{\sum }I\left(m,n\right)K\left(i-m,\;k-n\right) \end{array}$$

Whereby the image I is convolved with filter kernel K. Due to this convolution action, the CNN is capable of accounting for local connectivity, allowing for the capability to detect features invariant of location in the image. A nonlinear activation function is typically applied after convolutions. Typical activation functions utilized are the hyperbolic tangent function, the sigmoid function or the rectified linear unit (ReLU). Of interest to this study is the ReLU activation function. The ReLU function may be represented mathematically as:


(2) $$\begin{array}{l} ReLU:f\left(x\right)=\max\left(0,x\right) \end{array}$$

Of the activation functions listed above, the ReLU activation function is particularly popular due to its simplicity, as well as faster convergence and higher computational efficiency in comparison to the other activation functions mentioned above (Nair and Hinton, 2010). In addition, the ReLU function stands out due to its capability in mitigating the vanishing gradient problem, which was validated in the work by Ertam and Aydonn, where the group tested several activation functions for classification tasks. It was found that the ReLU performed the best in comparison, with an accuracy of 98.43% (Ertam and Aydin, 2017).

Pooling layers are generally inserted between convolutional blocks to reduce the image size and the number of variables to be calculated while maintaining descriptions of features. Rather than the precise feature locations outputted by the convolutional layer, subsequent operations are performed on the summarized features from the pooling layer, allowing for the network to be more robust to variations in feature locations. Popular pooling methods involve max pooling and average pooling, in which the maximum or average value within a local region is selected to represent its entirety. The fully connected layers refer to layers of densely connected neurons, serving to reduce the 2-dimensional features extracted into 1-dimensional vectors. In general, with classification tasks, the last layer of the fully connected layer is composed of neurons, each of which represents the probability of a certain class being the correct selection.

The learning process of the CNN model is facilitated with training algorithms. Optimization of the network is typically performed with gradient methods, such as gradient descent or stochastic gradient descent (SGD). Algorithms such as Adagrad (Duchi et al., 2011) and RMSProp perform SGD while simultaneously altering learning rates adaptively, boasting increased robustness and capabilities for dealing with sparse data. In this study, the adaptive moment estimation optimizer, or Adam optimizer, was employed (Hinton et al., 2012). Initially developed by Kingma and Ba, the Adam optimizer is another variant SGD optimizer capable of altering learning rates adaptively (Kingma and Ba, 2014). This method is especially efficient when dealing with models with large numbers of parameters and has been shown to outperform popular optimizers such as Adagrad and RMSProp in terms of accuracy and training cost (Chen et al., 2018).

## 4. Results and discussion for transfer learning models employed

As is common with various deep learning algorithms, CNNs rely heavily on annotated data. As briefly elaborated in the preceding section, CNNs generally acquire high-level features through their convolutional layers via learning algorithms, and subsequently execute classification tasks utilizing their fully connected layers. However, to facilitate this process, the dataset under consideration must possess sufficient scale. This ensures that the deep learning algorithms can effectively capture the inherent patterns and distinctive attributes within the data. Transfer learning involves the retention of knowledge and insights acquired while addressing a specific problem. This accumulated knowledge is subsequently repurposed and applied to solve distinct yet related problems (Bozinovski and Fulgosi, 1976). This approach obviates the necessity for models to be trained entirely from scratch, leading to notable reductions in resource demands, computational requirements, data volume, variance, and training duration. In fields such as microbiology, characterized by challenges in data acquisition due to specialized equipment needs and labor-intensive processes, leveraging data augmentation and transfer learning can be particularly advantageous. In light of this context, consider the example of the DIBaS dataset, comprising a modest collection of 660 digital images. The dataset's limited size imposes constraints on the effectiveness and robustness of deep learning models, particularly if training were initiated anew. To address this challenge, apart from the data augmentation techniques expounded upon in section 3.1.2, the application of transfer learning emerges as a viable strategy to alleviate the dataset limitations.

### 4.1. Experimental setup

Models were built with the Python programming language, employing modules from the PyTorch and TorchVision libraries, which have gained great popularity in the research community as an open-source machine learning framework. All models were trained on the 0.82 GHz 2-core NVidia K80 GPU. The GPU used has a Random Access Memory (RAM) of 12GB. As specified in Section 3.1, the dataset employed is the Digital Image of Bacterial Species (DIBaS) dataset, published by Zieliński et al. in their study: “Deep learning approach to bacterial colony classification” (Zieliński et al., 2017). Data augmentations were performed as specified in Section 3.2. Data inputted are split, allocating 80% of the total augmented dataset as training data, and 10% of the dataset as validation dataset, and 10% as testing dataset.

Evaluation of the performance of transfer learning models were determined based on several metrics. From literature, the most common metrics for evaluation of machine learning algorithms are parameters such as accuracy, precision, sensitivity (also known as recall), and F1 score (Fawcett, 2006). These parameters are usually calculated or derived from the confusion matrix: a table that plots the algorithm's confidence in the predicted classification versus the true classification of the test dataset. The confusion matrix allows for the visualization of algorithm performance. Evaluations based on the function for receiver operating characteristics curve and area under the curve are also popular, but are mainly employed for binary classification. With respect to the DIBaS dataset, with its 33 classes, an approach toward the evaluation of multiclass classification was deemed to be more suitable. The metrics of accuracy, precision, sensitivity, and F1 score were applied. Metrics for evaluation may be calculated from the Confusion matrix based on counts of True Positives (TP), True Negatives (TN), False Positives (FP) and False Negatives (FN), where positive or negative denotes the models prediction of class, and True or False denotes whether the model is correct in its prediction of class.

Accuracy may be represented as the fraction of correct predictions that the model has made, amongst all predictions made. It may be represented mathematically as:


(3) $$\begin{array}{l} Accuracy=\frac{TP+TN}{TP+TN+FP+FN} \end{array}$$

Precision describes the fraction of correct predictions, in the case that the model prediction is positive. It may be represented as the fraction of TP overall positive predictions. Mathematically, precision may be represented as:


(4) $$\begin{array}{l} Precision=\frac{TP}{TP+FP} \end{array}$$

Recall describes the rate of correct positive predictions, in the case whereby the true value is positive. It is represented as the fraction of actual true positive instances that the model correctly classifies, amongst all instances that are true positives. Mathematically, the formula for recall is given as:


(5) $$\begin{array}{l} Recall=\frac{TP}{TP+FN} \end{array}$$

The F1-score is defined as the harmonic mean of recall and precision. The metric is employed as an objective method to compare the performances of classifiers. It is usually desirable to maximize both recall and precision. This is typically difficult in practice, due to the computing nature of precision and recall. The F1 score provides a metric that effectively combines the recall and Precision metrics to determine the performance of the model objectively. Mathematically, the F1 score is given as:


(6) $$\begin{array}{l} F1=2^{*}\frac{Precision\;\times \;Recall}{Precision\;+\;Recall} \end{array}$$

### 4.2. Pre-trained architectures analyzed in comparative study

**Figure 3:**
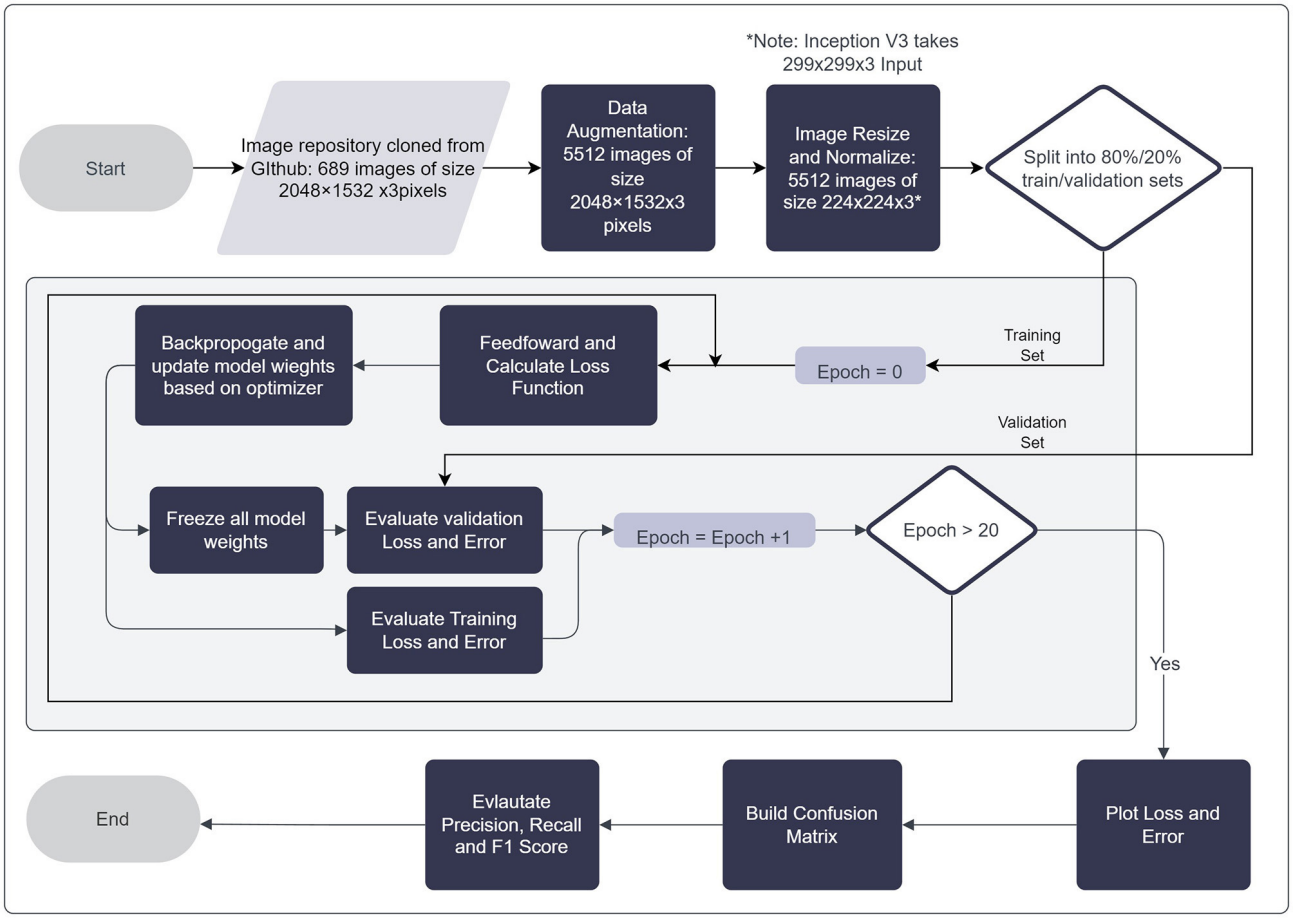
Flowchart of general training process for transfer learning models examined.

All models employed were trained on the ImageNet Dataset, a large, publicly available repository of more than 14 million images, of more than 20,000 different classes. All images are manually annotated with labels associated with objects that the image depicts. Models were initialized with their pre-trained weights from the ImageNet Dataset (Deng et al., 2009), then allowed to update their respective weights in accordance with features learned from the DIBaS dataset. Due to the amount and variety of data made available in the ImageNet dataset, various high-level features may be learned and re-applied to the visual recognition of bacteria species based on their physical features. Optimal hyper-parameter initialization is key to the performance of a neural network. Hyper-parameters such as batch size, learning rate and the number of epochs were selected with a manual search. In summary, batch size was varied from a range of 16 to 64, and the learning rate was varied from 0.00005 to 0.00015. A summary of the models employed and their associated documentation are provided in Table 5. The parameters used to tune the model examined with respect to the DIBaS dataset are summarized in Table 6. Additionally, the process by which models undergo training on the DIBaS dataset is summarized in the flowchart depicted in Figure 3.

**Table 5:** Transfer learning models reviewed in study.

**Model name**	**Associated research paper**	**Year published**	**Reference**
AlexNet	ImageNet classification with deep convolutional neural networks	2012	Krizhevsky et al. (2012)
GoogLeNet	Going deeper with convolutions	2014	Szegedy et al. (2015)
Inception V3	Rethinking the inception architecture for computer vision	2015	Szegedy et al. (2016)
Visual geometry group networks	Very deep convolutional networks for large-scale image recognition	2015	Simonyan and Zisserman (2014)
ResNets	Deep residual learning for image recognition	2016	He et al. (2016)
DenseNets	Densely connected convolutional networks	2018	Huang et al. (2017)

**Table 6:** Hyperparameters employed for transfer learning models.

**Model name**	**Initial learning rate**	**Batch size**
AlexNet	0.00008	128
GoogLeNet	0.0001	32
Inception V3	0.0001	32
VGG16	0.0001	64
VGG19	0.0001	64
ResNet-18	0.0001	64
ResNet-34	0.00008	72
ResNet-50	0.0001	64
ResNet-152	0.00008	32
DenseNets-121	0.0001	32
DenseNets-161	0.0001	16
DenseNets-169	0.0001	16
DenseNets-201	0.00008	16

#### 4.2.1. AlexNet

The AlexNet architecture, introduced by Krizhevsky et al. (2012), is a CNN design that underwent training using the ImageNet dataset. Its structure encompasses five convolutional layers with accompanying max-pooling operations, succeeded by three fully connected layers. The activation function employed is ReLU, chosen for its observed rapid convergence when compared to alternative functions such as hyperbolic tangent and sigmoid activations. In contrast to its predecessor, the LeNet-5 model, AlexNet integrates additional convolutional and pooling layers to achieve data normalization, rendering it adept at processing substantial data volumes. However, this stacked architecture implies an elevated parameter count, leading to computational inefficiencies relative to other explored models.

In this investigation, the final layer of the AlexNet Model's classifier is adjusted to yield 33 activations. Experimental findings indicate the model's generalizability, achieving a validation accuracy of 95.74% and a training accuracy of 98.82%. The marginal ~3% variance between training and validation performance can be attributed to the model's familiarity with training data and its relative novelty with respect to the validation dataset. Initial performance was modest, with validation and training accuracies at 15.70% and 34.45% respectively, but significant growth occurred in the first six epochs, culminating in a validation accuracy of 89.04%. Subsequent epochs yielded diminishing returns, particularly after the ninth epoch, where validation accuracy plateaued at 94.52% and validation loss reached 0.0014. Minimal improvements (<1%) were observed thereafter, indicating a slowdown in the acquisition of new features.

#### 4.2.2. Inception networks

**Figure 4:**
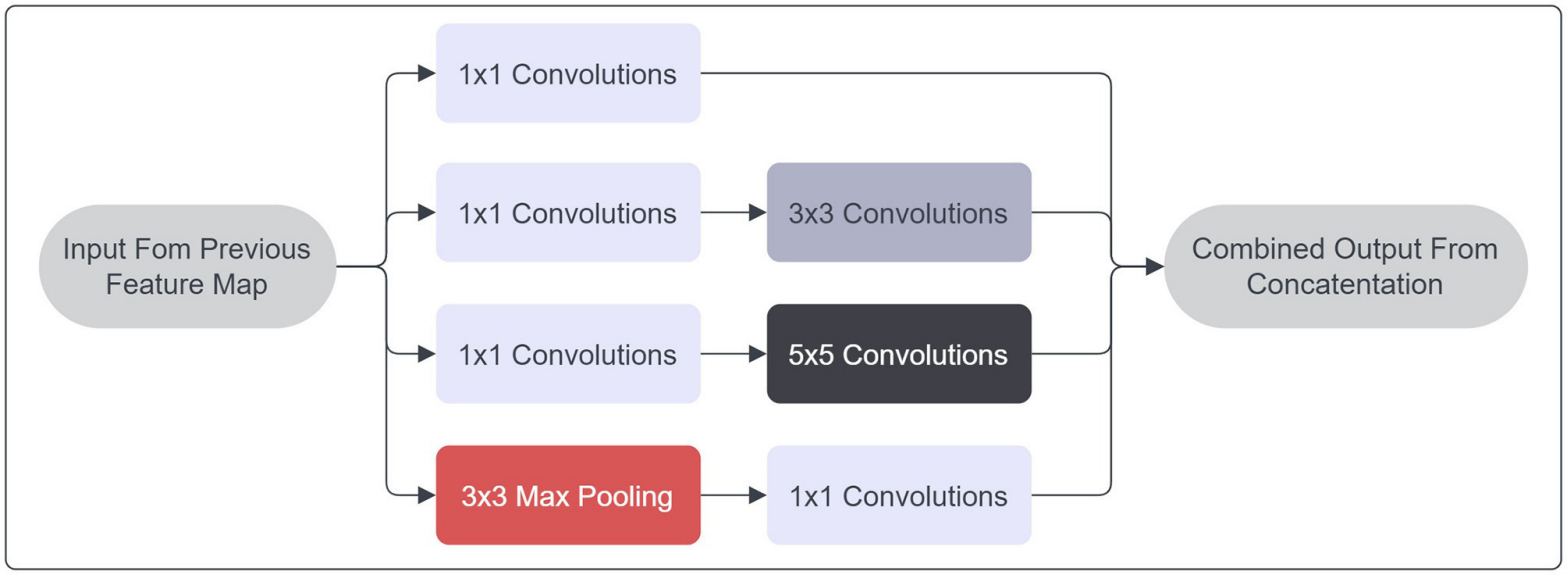
Inception module architecture.

The architecture GoogLeNet, referred to as Inception V1, is predicated upon the work outlined in the research publication titled “Going Deeper with Convolutions” authored by Szegedy et al. (2016). This neural network comprises a total of 27 layers, integrated with max pooling operations. A notable contribution of this study is the introduction of the inception module paradigm. The integration of increased depth into deep neural networks frequently engenders challenges related to performance, notably encompassing concerns such as overfitting, as well as the vanishing and exploding gradient phenomena. Additionally, the utilization of stacked convolutional layers tends to exhibit suboptimal resource utilization in terms of memory allocation and computational efficiency. The innovation of the Inception Module, as devised by Szegedy et al., mitigates these challenges by employing a configuration in which multiple filters of distinct dimensions convolve across a singular layer. The schematic depiction of an Inception Module's fundamental structure is provided in Figure 4.

By employing 1x1 convolutional filters, a reduction in input dimensionality is achieved, thereby enhancing the computational efficiency of subsequent operations. Modulating the filter size imparts an additional advantage of enabling the network to discern features across varying scales. In lieu of augmenting depth, the inception model opts for lateral expansion. Each convolutional stratum is accompanied by the application of a ReLU activation function. The outcomes yielded by diverse filters are subsequently amalgamated to form the outputs of the inception module. The GoogLeNet framework integrates nine such modules throughout its architecture, succeeded by a global average pooling layer. The inclusion of average pooling layers serves to supplant the conventional fully connected stratum in standard CNNs. Contrary to the conventional practice of channeling feature outputs through a fully connected layer, the mean value of each feature map undergoes direct processing through the SoftMax classifier function. This strategic shift confers the advantageous attribute of heightened resilience against overfitting by virtue of the absence of parameter modulation. Furthermore, the adaptability of convolutional networks to this classification approach is enhanced, given its establishment of a discernible correlation between feature maps and anticipated classes. In totality, Szegedy et al. have substantiated the superior performance and efficiency of this architecture in comparison to the conventional stacked CNN counterparts.

For this study, the output of the final classifier layer was modified to have an output layer consisting of 33 neurons, corresponding to the probability of each species of bacteria being identified. The GoogLeNet architecture performed significantly better than that of AlexNet, with the model converging to a test accuracy of 98.63%. Initial estimates in the first epoch are also much more accurate with the GoogLeNet model, at 80.15% test accuracy. This high initial estimate may be attributed to various factors. One possible explanation is that the initial weights imported from training on the ImageNet dataset, when arranged in the GoogLeNet model, are more suitable for classifying data from the DIBaS dataset. Improvements to results slowed significantly near the 5th epoch, with further epochs demonstrating marginal improvements (<1%). The GoogLeNet model also features a significantly smaller gap between test and training accuracies and losses, with both converging to ~99%.

Szegedy et al. made substantial enhancements to the existing network architecture by introducing Inception V3, as documented in their paper titled “Rethinking the Inception Architecture for Computer Vision” (Szegedy et al., 2015). Several novel techniques were introduced to augment the Inception V3 model's capabilities. To address the computational demands posed by large convolution filters, Szegedy et al. proposed a strategy to factorize larger filters into smaller ones. In employing this approach, equivalent coverage to that of larger filters can be achieved at a reduced computational cost. For instance, within the Inception V1 model, the previously utilized 5-by-5 filter was replaced with two 3-by-3 filters, resulting in a notable reduction in trainable parameters per filter. To further mitigate computational load, the Inception V3 model incorporated the use of asymmetrical filters. This approach involves replacing an n-by-n filter with a combination of an n-by-1 and a 1-by-n filter. The integration of batch normalization was another enhancement introduced in the Inception V3 model. In addition to these advancements, the paper also introduced grid size reduction as an alternative technique for dimensionality reduction, complementing the conventional employment of pooling layers. This involved incorporating parallel convolutional layers in tandem with the pooling layer and subsequently concatenating the resulting output feature maps, akin to the architecture of Inception Networks.

The Inception V3 model shared similar performance to the V1 model, with a test accuracy of 98.18%. Similar to GoogLeNet, the re-use of weights from the ImageNet database yielded very high initial accuracy with the DIBaS dataset, as high as 80.78% on the first epoch. The rate of convergence was also similar, with improvements in accuracy becoming marginal (<1%) at around the 5th epoch. The additional complexity introduced, however, with respect to the Inception V1 model, did not appear to contribute to any further improvements in performance of the model.

#### 4.2.3. Visual geometry group networks

The Visual Geometry Group Network (VGGNet), developed by Simonyan and Zisserman, was designed for application to the ImageNet Dataset, as discussed in their work titled “Very Deep Convolutional Networks for Large-Scale Image Recognition” (Simonyan and Zisserman, 2014). This study scrutinizes two specific architectures: VGG16 and VGG19 models. Both of these networks utilize ReLU activation functions alongside max pooling. Notably, these models prioritize the incorporation of smaller convolutional filters with increased depth, as opposed to a solitary larger filter. This design philosophy shares parallels with the Inception networks, where the adoption of smaller filters contributes to reduced trainable parameters and a heightened number of weight layers. Empirical evidence provided by Simonyan and Zisserman underscores the improved performance and computational efficiency of this configuration compared to architectures like AlexNet, which relies on larger 7-by-7 filters. VGG16 features 13 convolutional layers, followed by three fully connected layers, whereas the VGG19 architecture employs the use of 16 convolutional filters with three fully connected layers. In the context of this investigation, alterations were exclusively applied to the final layer to introduce 33 output activations. It is worth noting that the architecture of VGG exhibits a configuration characterized by cascaded convolutional layers, resulting in a higher parameter count compared to the alternative architectures examined.

The complex nature of these systems becomes apparent through their performance evaluation using the DIBaS dataset. The heightened complexity resulted in extended training durations and greater utilization of GPU RAM space. The performance of the VGG16 model is akin to that of the Inception networks, converging to a final test accuracy of 97.34% and loss of 0.015. The initial accuracy of the first epoch is 79.96%. For the VGG19 structure, the additional convolutional layers did not appear to aid in the effective feature extraction and learning of the network, as the final accuracy converged to a value of 97.26%. The initial accuracy was also lower at 62.38%, although this may be attributed to the weight parameters from the ImageNet database within the VGG19 network not being as suitable for the DIBaS dataset.

#### 4.2.4. Residual networks

With the development of neural networks consisting of additional layers, an interesting phenomenon to note is that, as depth approaches a certain threshold, the performance of the network ceases to increase. Instead, the performance of very deep neural networks is, on occasion, lacking behind their shallower counterparts. This phenomenon has been attributed to various speculated causes, such as the activation function employed, overfitting, or the vanishing gradient problem. He et al. (2016) introduced a solution to the declining performance of neural networks with depth, through their introduction of residual, or skip connections (He et al., 2016). By means of these residual connection blocks, the information emanating from preceding layers is incorporated into successive layers. This enables a more unhindered transmission of information throughout the network, thereby enabling deeper layers to achieve performance parity with shallower layers. The structure of a residual block may be represented mathematically as:


(7) $$\begin{array}{l} x_{l}=H_{l}\left(x_{l-1}\right)+\;x_{l-1} \end{array}$$

**Figure 5:**
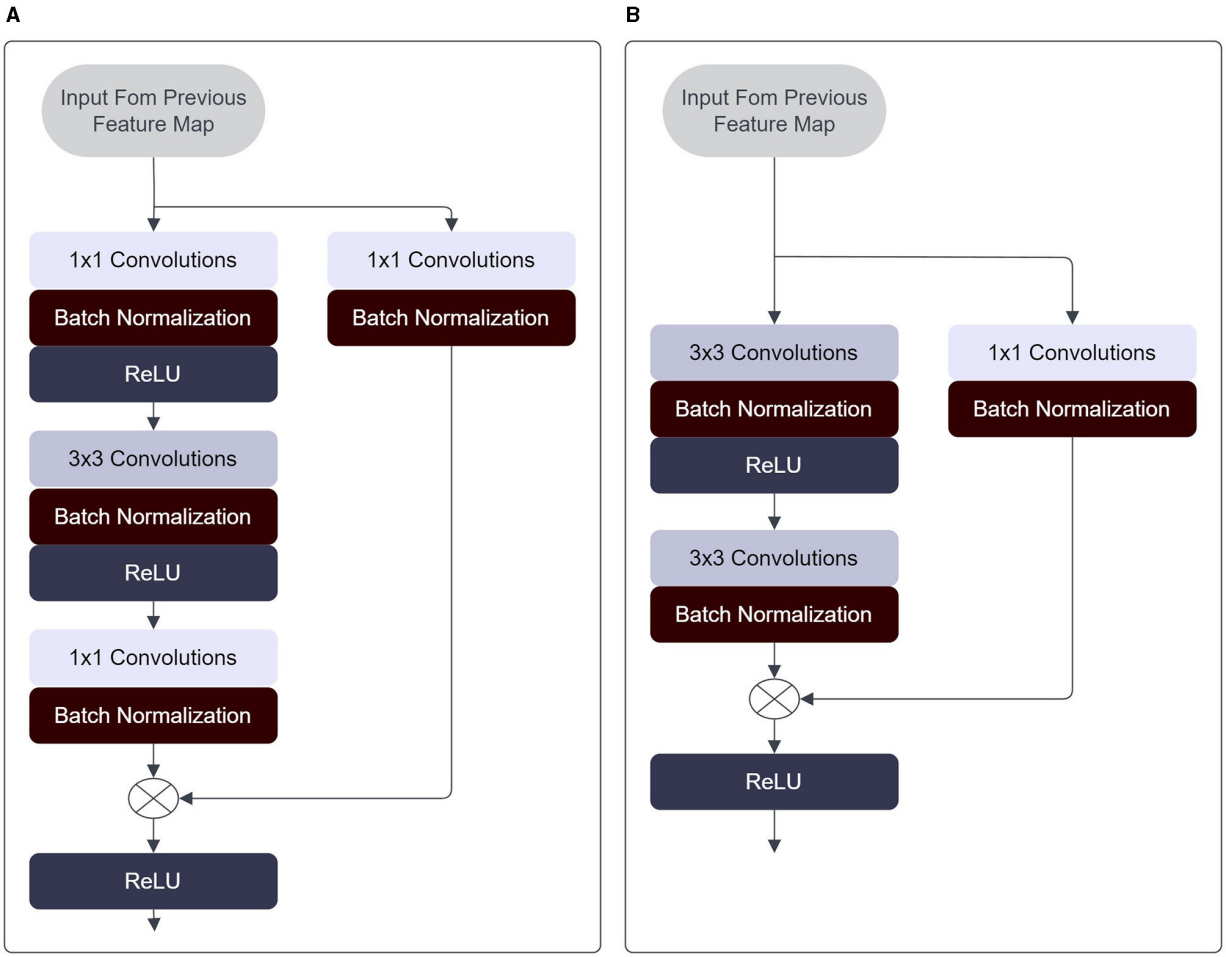
**(A)** Architecture of residual block employed in the ResNet-18 and ResNet-34 models. **(B)** Architecture of residual block employed in the ResNet-50 and ResNet-152 models.

Whereby the inputs of layer (x_l_) are comprised of previous layer information (x_l − 1_) combined with the previous layer information parsed through some transfer function (H) via element-wise addition. The function H maps the input of x to some output, based on the set of weights applied and activation functions, through the convolution operation typical of CNNs. In this fashion, much deeper neural networks may be trained without suffering losses in accuracy. In the research conducted by He et al., it has been established that Residual Networks exhibit superior convergence speed and precision in comparison to conventional neural networks with equivalent depth. Notable distinctions are present within the structural composition of residual blocks employed in the ResNet architectures. Refer to Figure 5A for a visualization of the residual blocks used in ResNet-18 and ResNet-34 models, while Figure 5B illustrates the residual blocks utilized in ResNet-50 and ResNet-152 models.

In this investigation, the sole alteration applied involved adjusting the quantity of activation neurons within the output layer. Overall, the ResNet framework exhibited notably swift convergence rates, wherein a majority of models accomplished approximately 90% accuracy on the testing dataset and a loss of around 0.005 following a solitary epoch. In comparison with the stacked convolutional layers of the VGG model, the Residual networks achieved similar results with fewer training. With the ResNet-152 model, the initial test accuracy was the highest of all models tested, at 93.11%, and ultimately converging to a final accuracy of 98.82% over the course of 20 epochs. The other architectures of residual networks achieved less notable results, with an accuracy of 97.88%, 98.23% and 98.12% for the ResNet-18, ResNet-34 and ResNet-50 respectively.

#### 4.2.5. Densely connected networks

DenseNets were formulated by Huang and colleagues in their research titled “Densely Connected Convolutional Networks” (Huang et al., 2017). Analogous to the approach seen in Residual Networks, the paper's authors also adopted the strategy of integrating inputs from preceding layers. However, in contrast to the summation of output feature maps, each individual layer within a DenseNet generates an output through concatenation of the outputs from all preceding layers. As opposed to the ResNet, this relation may be represented as:


(8) $$\begin{array}{l} x_{l}=H_{l}\left(x_{0},\;x_{1},\;\ldots ,x_{l-2},x_{l-1}\right) \end{array}$$

**Figure 6:**
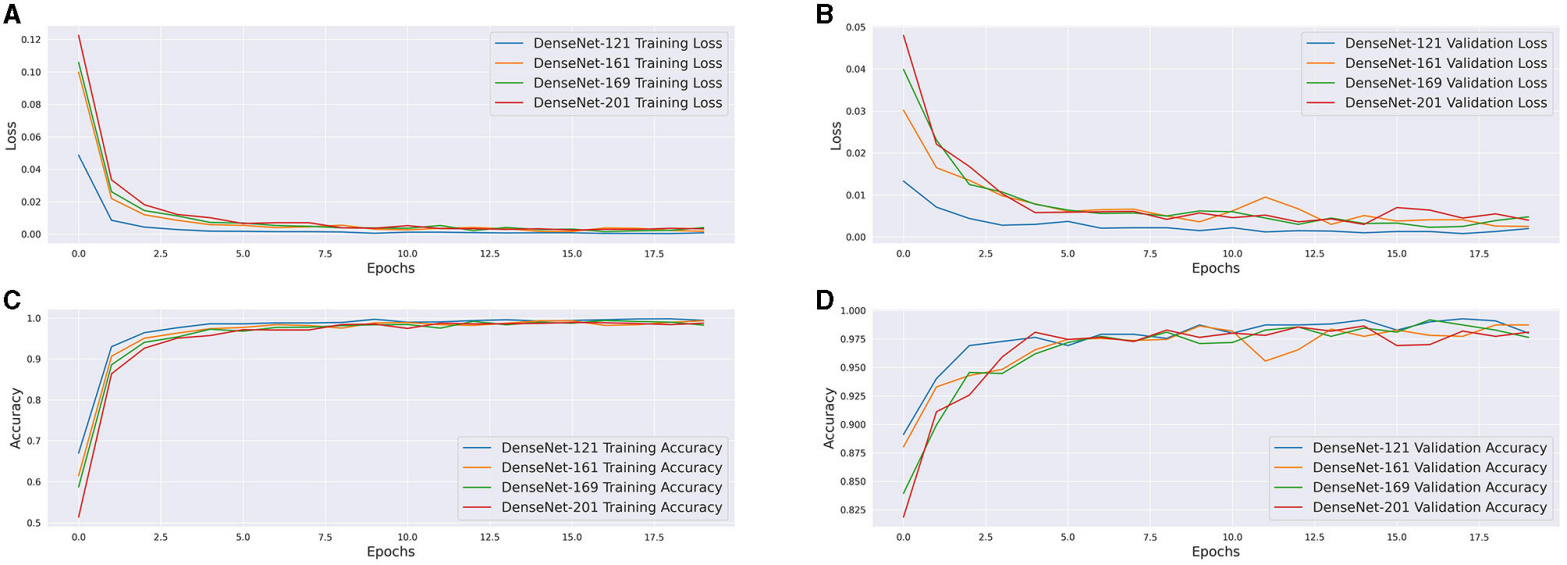
**(A)** Training Loss of DenseNet architectures. **(B)** Validation Loss of DenseNet architectures. **(C)** Training Accuracy of DenseNet architectures. **(D)** Validation Accuracy of DenseNet architectures.

Whereby each dense layer (x_l_) is represented as a function of all prior layers. The motivation behind DenseNets is to address the challenge posed by the vanishing gradient problem, thereby enhancing accuracy. In terms of structure, DenseNets are constituted by dense blocks and transitional layers. Each dense block is comprised of several collections of batch normalization, ReLU, and convolution layers. As previously mentioned, within every dense block, each grouping of batch normalization, ReLU, and convolution layers are densely interconnected with all preceding groupings. It is important to highlight that inside the dense block, the dimensions of all feature maps are consistent, thereby enabling subsequent convolutional operations. Transitional layers are responsible for carrying out the down-sampling or pooling task. Every transitional layer is composed of a batch normalization step, a 1-by-1 convolution, and a 2-by-2 average pooling operation. The loss and accuracy curves of each model are depicted in Figure 6. The presence of densely connected convolutional layers in the DenseNet framework enhances the capacity of successive layers to acquire a broader range of dataset features in a more streamlined manner. The interconnectedness of these layers ensures that each feature map is employed by all subsequent layers within a given dense block. Consequently, the DenseNet architecture demonstrates a heightened proficiency in assimilating intricate features, all the while curtailing the acquisition of unnecessary and indistinct features. Notably, owing to the limited dataset size, the practice of reusing features seems to have notably augmented performance and convergence outcomes.

The DenseNet-121 model, amongst the models tested, boasted the highest accuracy, with a test accuracy converging to 99.08%. Convergence was also exceptionally fast, with marginal (<1%) improvements to accuracy and loss occurring as early as the 3rd epoch. Overall, the DenseNet-121 model generalized the best to the new dataset provided, although this may be attributed to the sample of data available for analysis, whereby simpler models are favored. The other DenseNet architectures performed fairly well with accuracies of 98.17%, 98.40%, and 98.57% for DenseNet-161, DenseNet-169 and DenseNet-201 respectively.

### 4.3. Analysis of results

**Figure 7:**
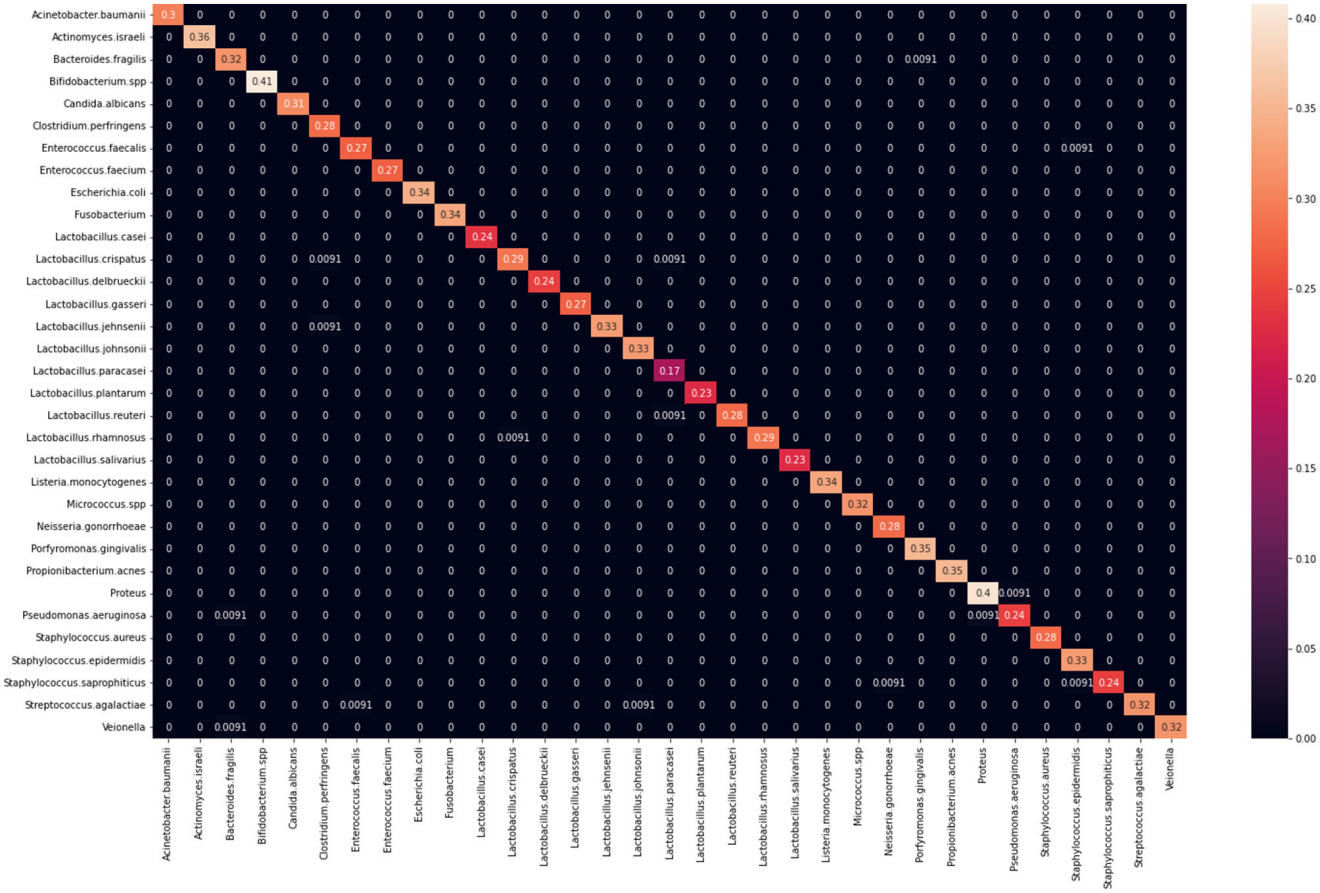
Confusion matrix derived for the DenseNet-121 model.

A summary depicting the performance of the diverse networks enumerated in the preceding section can be located within Table 7. The assessment of performance involved the consideration of accuracy, precision, recall, and F1 Score. Furthermore, the depiction of the confusion matrix for the most proficient model is presented in Figure 7. The computational time and parameter count requiring training for each model are subjected to analysis, as presented in Table 8. The training of all models spans 20 epochs, facilitating a basis for comparison concerning the duration allocated to training and validating the data. The study's outcomes reveal modest standard deviations due to the relatively limited size of the training dataset. Accuracy metrics span from 95.57% to 99.03%, precision ranges from 95.90% to 99.12%, recall varies between 95.55% and 99.09%, and the F1 score ranges from 95.56% to 99.08%. Most models have demonstrated commendable adaptability to the novel dataset, with the least accurate being AlexNet at 95.57%. Notably, among the assessed models, VGG19, possessing the highest complexity with 139,705,441 parameters, demonstrates inferior accuracy compared to all other models, barring just AlexNet. This observation could potentially be ascribed to the uncomplicated nature of the dataset. The dataset, post augmentation, comprises a mere 5940 images, possibly rendering more straightforward models more suitable. The substantial parameter count within the VGG19 model might be considered surplus when confronted with the expanded DIBaS dataset.

**Table 7:** Tabulated accuracy, precision, recall and F1 scores for models examined.

**Model**	**Top-1 accuracy**	**Precision**	**Recall**	**F1 score**
AlexNet	95.74%	95.90%	95.55%	95.56%
GoogLeNet	98.63%	98.86%	98.82%	98.82%
Inception V3	98.18%	98.67%	98.64%	98.63%
VGG16	97.34%	97.69%	97.55%	97.47%
VGG19	97.26%	98.79%	98.73%	98.73%
ResNet18	97.88%	98.05%	97.91%	97.92%
ResNet34	98.23%	98.33%	98.28%	98.28%
ResNet50	98.12%	97.44%	97.10%	97.10%
ResNet152	98.87%	98.89%	98.82%	98.82%
DenseNet121	99.08%	99.06%	99.00%	98.99%
DenseNet161	98.17%	99.12%	99.09%	99.08%
DenseNet169	98.40%	98.87%	98.82%	98.82%
DenseNet201	98.57%	98.96%	98.91%	98.90%

**Table 8:** Tabulated training time and the number of parameters for models examined.

**Network name**	**Number of parameters to be trained**	**Time**	**Parameters trained per second**
AlexNet	57,139,041	47 min 7 s	20,211
GoogLeNet	6,194,625	49 min	2,107
InceptionV3	27,194,297	1 h 9 min 51 s	6,488
VGG16	134,395,745	1 h 4 min 49 s	34,557
VGG19	139,705,441	1 h 11 min 23 s	32,618
ResNet18	11,689,512	48 min 24 s	4,025
ResNet34	25,557,032	50 min 49 s	8,382
ResNet50	25,557,032	53 min 51 s	7,909
ResNet152	60,192,808	1 h 31min 25 s	10,982
DenseNet121	8,011,889	1 h 7 s	2,221
DenseNet161	28,714,033	1 h 21min 5 s	5,902
DenseNet169	14,182,513	1 h 6min 22 s	3,561
DenseNet201	20,046,961	1 h 12min 13 s	4,626

Among the various models assessed, it becomes evident that the DenseNet-121 model exhibited the highest level of accuracy. When considering precision, recall, and F1 score metrics, the DenseNet-161 model displayed superior performance compared to the other models. Specifically, the metrics recorded values of 99.12%, 99.09%, and 99.08%, respectively. However, the accuracy achieved by this model was slightly lower, standing at 98.17%. Notably, the dataset maintains a reasonably balanced distribution across its classes, with approximately 20 images representing each bacterial species category in the original dataset. In this context, accuracy emerges as the more reliable predictor of model effectiveness. Based on the examination of 13 models, it can be deduced that the DenseNet-121 model emerges as the most favorable architecture concerning the DIBaS dataset. In the realm of performance versus computational cost, the GoogLeNet or Inception V1 model showcased the least parameter count, totaling 6,194,625 trainable parameters within the model. Notably straightforward, this model also exhibited the swiftest training time, requiring only 49 minutes and 0 seconds to complete 20 epochs. Despite its simplicity, the model managed to achieve results on par with the top-performing DenseNet-121 model, converging toward a test accuracy of 98.63%.

In the context of the DIBaS dataset (Digital Image of Bacterial Species), which comprises 660 images encompassing 33 distinct genera and species of bacteria, it is noteworthy that transfer learning algorithms, specifically DenseDets, have exhibited occasional misclassification of images, despite achieving an overall classification accuracy of 99.12%. This intriguing observation raises questions about the underlying factors contributing to such misclassifications within this specific dataset. Several potential explanations can be considered to elucidate these phenomena. Firstly, it is crucial to acknowledge that DIBaS presents a unique set of challenges due to the diversity and intricacy of bacterial species represented. These challenges include variations in bacterial morphology, staining techniques, and imaging conditions, which may introduce subtle but significant differences across images. Transfer learning algorithms like Densenets are often pretrained on large-scale datasets with different statistical properties, potentially leading to a domain gap between the source and target datasets. As a consequence, the model may struggle to adapt effectively to the specific characteristics and nuances of the DIBaS dataset. Furthermore, the presence of rare or atypical bacterial species within DIBaS may pose challenges for transfer learning algorithms. Pretrained models may not have encountered such rare instances during their training on general datasets, resulting in a lack of representative knowledge for accurate classification. These rare species could be particularly susceptible to misclassification due to their limited presence in the training data.

Among the misclassifications observed in the DIBaS dataset, it is noteworthy that some of the most prominent errors pertain to distinguishing between images of Acinetobacter baumannii and Pseudomonas aeruginosa, as well as Lactobacillus gasseri and Bacteroides fragilis. These specific misclassifications may be attributed to several dataset-related factors and image characteristics.The challenges in discriminating between Acinetobacter baumannii and Pseudomonas aeruginosa could be attributed to their morphological similarities, particularly when observed under varying imaging conditions and staining techniques. Both species can exhibit similar rod-shaped or coccobacillary forms, making it challenging for a model to capture the subtle differentiating features. The limited availability of diverse images capturing distinct variations of these species in the DIBaS dataset may contribute to the model's difficulty in effectively distinguishing between them. Similarly, the misclassifications involving Lactobacillus gasseri and Bacteroides fragilis may arise from their overlapping characteristics in terms of cellular shape and arrangement, which may be challenging to discern in microscopic images. These genera share similarities in cellular morphology, such as being gram-positive rods or cocci, further complicating the classification task. The relative scarcity of images representing unique variants of these species within the DIBaS dataset may hinder the model's ability to generalize accurately to distinguish them.

In summary, while Densenets demonstrate comparatively high performance on the DIBaS dataset, the occasional misclassifications observed highlight the importance of understanding the dataset-specific challenges and intricacies. Factors observed above may collectively contribute to the misclassification phenomenon and warrant further investigation and potential dataset-specific fine-tuning strategies to enhance the performance of transfer learning algorithms on the DIBaS dataset.

### 4.4. Limitations and future work

With respect to the dataset employed, one limitation of our proposed method is that, although it demonstrated promising results for the Digital Image of Bacterial Species (DIBaS) dataset used in this study, its performance may exhibit variability when applied to different datasets. This variability can be attributed to the unique characteristics and challenges presented by various datasets, which might not align with the specific features of the DIBaS dataset. As a potential avenue for future work, our study could be extended to encompass a diverse range of datasets beyond the Digital Image of Bacterial Species (DIBaS) dataset. This extension would allow us to assess the adaptability and robustness of our proposed method across datasets with varying characteristics and challenges, thereby addressing the limitation of potential performance variability highlighted earlier. By exploring multiple datasets, we can gain a more comprehensive understanding of the method's generalizability and effectiveness in different contexts.

Furthermore, some limitations in generalizability exist for the study performed. Analysis was mainly performed on an initial dataset of 689 images. Although data augmentations have been performed, the augmented dataset is only comprised of 5,512 digital images. Additionally, the transfer learning models examined are built upon the ImageNet database, which consists of a wide variety of images of real-world objects. Although many image features may be learned with the ImageNet database as a foundation, the dataset may not be specialized in bacteria species identification. Due to the limited dataset of bacterial species available, the transfer learning model may not be optimized for the task of bacterial identification. For the purposes of training a deep learning architecture, a higher robustness and accuracy may be achieved with higher quantities of training data. Future work in this area may involve a more refined strategy of data augmentation or the construction of an alternate dataset specialized for the application. Furthermore, models explored in this study rely mainly on altering the structures and connections of traditional CNNs. As the field of deep learning and image processing evolves, innovations on the structure and layout of deep learning networks are already prevalent with the integration with image processing strategies such as of Vision transformers. As newer, more refined algorithms are developed, a comparative analysis may prove valuable in the determination of optimality with regards to industry specific applications.

## 5. Concluding remarks

In conclusion, this study provides a comprehensive overview of modern machine learning methodologies applied within the healthcare context, specifically concentrating on their role in preventing infectious diseases and identifying bacterial entities. The research commences with a concise examination of current machine learning algorithms utilized in healthcare, underscoring their significance in the realm of bacterial species diagnosis and classification. Noteworthy is the thorough literature analysis encompassing a wide array of studies employing machine learning and deep learning algorithms for microbial diagnostics. Various methodologies for categorizing bacterial micro-bodies using machine learning approaches were examined. Among the 29 instances of literature surveyed, 7 (24.14%) focused on classical machine learning algorithms applied to bacterial identification. Additionally, 9 (31.03%) works explored the implementation of deep learning methods, including the multilayer perceptron model and convolutional neural networks. Furthermore, 11 (37.93%) studies incorporated transfer learning by leveraging pre-trained convolutional neural network architectures along with data augmentation. The analysis of the compiled literature reveals the predominance of convolutional neural networks as the preferred deep learning model for image classification, yielding favorable outcomes. The study also assesses the efficacy of current transfer learning algorithms when dealing with limited data samples, particularly in the context of microscopic bacteria image classification.

Particular emphasis is placed on Convolutional Neural Networks (CNNs), a pivotal choice for over a decade due to their autonomous feature extraction capabilities with minimal human intervention. A noteworthy trend in the field is the increasing utilization of transfer learning, repurposing pre-trained models for classifying microbial images. Importantly, this study goes beyond prior reviews by not only summarizing existing research but also by actively implementing and evaluating transfer learning algorithms for microbial detection, using the DIBAS dataset. This pragmatic approach adds a tangible dimension to the study, demonstrating the real-world effectiveness and limitations of these techniques. To assess the performance of different convolutional neural network architectures, pre-trained weights from the ImageNet database were employed. These models (totaling 13) were evaluated using the Digital Images of Bacterial Species dataset. The evaluation encompassed both statistical metrics and overall model complexity and efficiency. From a statistical standpoint, the DenseNet-121 model, boasting approximately 8 million trainable parameters, exhibited superior performance in the classification task. It achieved an accuracy of 99.08%, precision of 99.06%, recall of 99.00%, and an F1 score of 98.9%. Comparatively, the DenseNet-161 model outperformed its counterparts in terms of precision, recall, and the F1 score. While it achieved an accuracy of 98.17%, its precision reached 99.12%, recall was 99.09%, and the F1 score reached 99.08%.

## Author contributions

YW and SG contributed to conception and design of the study. YW organized the literature survey and wrote the first draft of the manuscript. All authors contributed to manuscript revision, read, and approved the submitted version.
